# Digital teaching after the pandemic – enriching diversity of teaching methods and freedom for inclination-oriented learning?

**DOI:** 10.3205/zma001507

**Published:** 2021-09-15

**Authors:** Martin R. Fischer

**Affiliations:** 1 LMU Munich, University Hospital, Institute of Medical Education, Munich, Germany

## Editorial

Regardless of the ongoing COVID-19 pandemic, digital teaching and examination formats should be used regularly and sustainably in medical curricula to an appropriate extent and according to site-specific possibilities. As a joint position paper of MFT and GMA states [1]: “The challenge here is to use the new digital formats as an important and curricularly fully creditable supplement to the established face-to-face teaching formats in order to support self-directed learning and the development of a knowledge base and to create free space for the essential application and discussion of what has been learned in the face-to-face teaching”. The personal and socially experiential dimension of learning, especially in the application- and patient-oriented formats of medical education, remain pandemic presence-independent. 

“However, the potentials for effective and efficient further development of digital teaching-learning methods have become clear through the pandemic-related experiences and require further critical and sustained analysis. Forms of digital teaching range from asynchronous formats to synchronous interactive formats to complex small group formats of collaborative learning. The advantage of asynchronous formats is the possibility of self-directed learning, which allows for different learning paces, repeated access and repetition, and individual focus based on knowledge gaps. Synchronous formats allow direct exchange between instructors and students in interactive learning phases, clarification of open questions and comprehension problems, and collaborative learning among students. They offer an alternative to parts of face-to-face teaching and can thus be a profitable supplement to previous face-to-face teaching methods. Great potential of digital formats is that students are well prepared for the necessary face-to-face teaching, for simulation-based teaching formats and for training in a clinical context.” [1] 

Blended learning concepts with well-coordinated digital offerings provide an important opportunity at medical schools for more individualized competency-based training in line with the current National Competency-Based Learning Objectives Catalog 2.0 (see [http://www.nklm.de]). They also allow flexible and organizationally low-threshold linking of courses for participants from different training and study programs to promote interprofessional teaching. In addition, they offer flexible options for disadvantage compensation in favor of impaired students. As a matter of principle, blended learning should be offered to students from all faculties in an appropriate quality and should be firmly anchored in the curricula. This also opens up new implementation possibilities for inclination-oriented courses on the part of the teachers, which can be offered across semesters within the framework of elective curricula with little organizational effort, independently of the specifications of the NKLM (“Teach whatever you want!”). This can counteract the fears of excessive schooling due to excessive specifications through learning objective catalogs such as the NKLM [2].

There still is a lot of work to do: “At the medical faculties, a sustainable infrastructure for digital teaching is required that is seamlessly linked to the IT infrastructure of the universities, university hospitals and academic teaching hospitals. This requires a coordinated and transparent procedure between these two IT worlds for the release of software solutions in accordance with data protection and usage laws. Lecturers should be able to rely on the usability of classic tools of digital teaching at both university and clinical computer workstations, such as campus management and learning management systems, video conferencing systems, and examination and evaluation environments in particular. Appropriate hardware and software requirements are to be created at designated workstations for the development and recording of asynchronously usable digital teaching and learning materials. Teachers should be able to prepare and process their media without technical hurdles, especially with the help of media servers with appropriate capacities.” [1] To this end, well-coordinated software packages and licenses with open source solutions should be strived for and – wherever possible – implemented and maintained across faculties [3]. 

“The technical requirements and needs of the students should be taken into account accordingly. For the didactically-conceptually and technically appropriate creation of digital teaching and learning materials, easily accessible and comprehensible training materials are required. These materials must be combined with a demand-oriented and sustainably offered training program for all teachers and teaching administrators. In addition, a support offer, e.g. in the form of student eScouts and eTutors as well as by the corresponding media departments, is important to ensure a sufficient quality standard of the teaching materials on the one hand and a secure, easy-to-find and sufficiently performant technical provision on the other hand.” [1]

For the economic use of digital teaching/learning resources, it is essential to make use of available offerings, some of which are publicly funded (e.g. Virtual University of Bavaria, see [https://www.vhb.org/en/]), both at the state level and at the federal level (e.g. LOOOPshare platform of the MFT [https://looop-share.charite.de/]). These should be expanded in a targeted and concerted manner. To this end, a sustainable national funding program based on the concept of the vhb is required, which differentiates between new creation and revision of digital teaching/learning resources and also rewards their support. Last but not least, it must be ensured that sufficient end devices that meet the performance requirements are available to all students, especially for digital examinations. While digital exam formats are widely and securely used in many faculties, remote exam formats are primarily an effective means of providing formative feedback. The legally compliant and fair provision of summative digital exams requires much further thought and implementation effort in terms of their legal and data security. In any case, however, the prerequisite for this is a resilient technical infrastructure.

This issue of the GMS Journal for Medical Education spans a wide range in terms of teaching methods and contexts with contributions on clinical face-to-face teaching in the form of rounds [4]) and suggestions for improving anamnesis techniques [5], contributions on simulation-based learning [6] and the use of VR technology [7] to digital trends in teaching [8]. The contributions on interprofessional teaching [9] and on measuring learning success with the help of formative examination formats [10] are also representative of the important and challenging development and integration work of these topics in the future with the help of teaching and examination formats in presence and in digital form.

## Competing interests

The author declares that he has no competing interests.
